# CT evaluation of lung infiltrates in the two months preceding the Coronavirus disease 19 pandemic in Canton Ticino (Switzerland): were there suspicious cases before the official first case?

**DOI:** 10.1007/s11547-022-01466-9

**Published:** 2022-03-05

**Authors:** Stefania Rizzo, Carola Catanese, Carla Puligheddu, Samantha Epistolio, Giulia Ramelli, Milo Frattini, Ricardo Pereira Mestre, Navarajah Nadarajah, Ermidio Rezzonico, Francesco Magoga, Lisa Milan, Filippo Del Grande, Luca Giovanella, Luca Ceriani

**Affiliations:** 1 Imaging Institute of Southern Switzerland (IIMSI), Ente Ospedaliero Cantonale (EOC), Via Tesserete 46, 6900 Lugano, Switzerland; 2 Facoltà Di Scienze Biomediche, Università della Svizzera italiana (USI), Lugano, Switzerland; 3 Istituto Cantonale Di Patologia (ICP), Ente Ospedaliero Cantonale (EOC), Locarno, Switzerland; 4 Service of Medical Oncology, Oncology Institute of Southern Switzerland, Ente Ospedaliero Cantonale (EOC), Bellinzona, Switzerland; 5 University Hospital of Zurich, Zurich, Switzerland

**Keywords:** Pandemic, COVID-19, Computed tomography, Canton Ticino

## Abstract

**Purpose:**

The main objective of this study was to assess the presence of pulmonary infiltrates with computed tomography (CT) appearance compatible with infection by coronavirus disease 2019 (COVID-19), in Canton Ticino in the 2 months preceding the first official case. Secondary aims were to compare the classification of infiltrates in the same time frame in 2020 and 2019; to compare the number of chest CT scans in the same period; to search for pathological confirmation of the virus.

**Materials and methods:**

Chest CT scans performed between January 1 and February 24 in 2019 and 2020 were collected and classified by COVID-19 Reporting and Data System (CO-RADS). Pathological presence of the virus was searched for when appropriate material was available.

**Results:**

The final cohort included 881 patients. Among the CO-RADS 3 and 4 categories, 30 patients had pneumonitis of unknown etiology. Pathological specimens were available in six patients but they were negative for COVID-19.

**Conclusion:**

Before the first official case of COVID-19 infection, in Canton Ticino there were about 30 cases of pneumonitis of uncertain origin, with CT appearance compatible with infection by COVID-19, but with no confirmation of the disease. The number of chest CT scans in the first two months of 2020 was > 12% compared to 2019.

## Introduction

Pneumonia caused by severe acute respiratory syndrome coronavirus 2 (SARS-CoV-2) infection emerged in Wuhan City, China, in December 2019. By February 11, 2020, the World Health Organization officially named the disease resulting from infection with SARS-CoV-2 as coronavirus disease 2019 (COVID-19). COVID-19 represents a spectrum of clinical manifestations that typically include fever, dry cough, and fatigue, often with pulmonary involvement, with an incubation period ranging between 0 and 24 days, with an average of 5–7 days [1].

Definitive diagnosis of COVID-19 is made using a reverse transcriptase-polymerase chain reaction (RT-PCR) assay, although reported sensitivities in clinical practice range between 42 and 83% depending on symptom duration, viral load, and test sample quality. On the other hand, CT scans, already routinely performed to make diagnoses, to assess complications and to guide imaging interventions [2, 3], are considered the best imaging modality to assess the presence of COVID-19-related pneumonitis when RT-PCR is negative, and the clinical suspicion is high [4, 5].

In early March 2020, the Dutch Radiological Society initiated a network with a dedicated working group that elaborated a COVID-19 Reporting and Data System (CO-RADS), to provide a level of suspicion for pulmonary involvement of COVID-19 based on the features seen on a non-enhanced chest CT. The level of suspicion increases from very low (CO-RADS 1) to very high (CO-RADS 5), rising to certain infection, confirmed by a positive RT-PCR (CO-RADS 6) [6]. This classification shows high performance in a setting with high prevalence of the COVID-19 disease.

When the pandemic was recognized in Europe, an epidemiological history of travels or residence in Hubei Province was present. The COVID-19 outbreak in Italy started in two places in the Northern part (Codogno, Lombardy, and Vo’ Euganeo, Veneto), and on February 25, 2020, 240 cases had been confirmed in Lombardy and 43 in Veneto [7]. Apolone et al. demonstrated an unexpected very early circulation of SARS-CoV-2 among asymptomatic individuals in Italy several months before the first patient was identified, clarifying the onset and spread of the COVID-19 pandemic and suggesting a possible reshape of its history [8].

Canton Ticino is located in the southern part of Switzerland, widely bordering the Italian region of Lombardy and many people travel across the two areas every day, mainly for work or for tourism. The first diagnosis of COVID-19 in Canton Ticino was ascertained on February 25, 2020. Before that date, the presence of the virus was not looked for, therefore we do not have data about the possible previous spread of the virus in this area.

To the best of our knowledge, no study has so far investigated the presence of COVID-19 pulmonary infiltrates before the first declared case in Southern Switzerland.

Therefore, the main objective of this study was to assess the presence of pulmonary infiltrates compatible with infection by COVID-19 (according to the CO-RADS classification) in a general population referred to the largest public hospital in Canton Ticino between January 1 and February 24, 2020. Secondary objectives were to compare the CO-RADS classification of infiltrates in the same time frame (January and February) in 2020 and 2019; to assess whether the number of chest CT scans performed to search for lung infiltrates, carried out in the first two months of 2020 was comparable to the same time period of 2019; to search for serological or pathological confirmation of the virus in patients classified as CO-RADS 3 or 4.

## Materials and methods

### Patient selection

Between June and August 2020, the radiology information system was used to retrieve the CT examinations performed between January 1, 2020 and February 24, in 2020 and in 2019 having the terms [“ground-glass” AND/OR “opacity” AND/OR “infiltrate” AND/OR “pneumonitis” AND/OR “consolidation”] mentioned in the relative report. Replicated scans, such as those performed as follow-ups of previously detected infiltrates or those including more than one of the search terms, but referring to the same patients, were furtherly excluded from evaluation. Accordingly, only one scan per patient (the one used for diagnosis) was evaluated according to CO-RADS.

According to the study protocol, we searched for clinical information in patients with CT findings categorized as CO-RADS > 3. The local Ethics Committee approved this retrospective study, asking for the informed consent of patients with CO-RADS classification > 3 in CT scans performed in 2020. Details of the study were sent to these patients, asking for their consent to the study and for the following information: travels in high-risk zones between January and February 2020; testing for an infection from COVID-19 (if yes, with indication of the date); testing for serological presence of antibodies to COVID-19. Later on, for included patients with CO-RADS 3 and 4 and availability of pathological specimens during the period under examination, specific written consent was requested for use of pathological specimens to screen for the presence of COVID-19.

Inclusion criteria were: presence of one or more of the search terms within the text of the CT report; CT scan available for review; consent of the patients as mentioned above. Exclusion criteria was: refusal of consent.

### CT Imaging

Examinations were randomly performed on the following CT scanners: Somatom Definition Edge (Siemens Healthcare); Brilliance iCT 256 (Philips Healthcare AG). The CT acquisition protocol was adapted to the clinical indication (e.g., with contrast medium if the clinical indication included the hypothesis of pulmonary embolism or in patients during follow-up for other pathologies; without contrast medium if clinical indication was for infiltrates or fever); multiplanar reconstructions were always made, as per institutional protocol.

### CO-RADS Classification

Three radiologists (SR, CC, CP, with > 15 years of experience in reading CT scans) rated the CT scans according to the CO-RADS classification. In case of discordance, the classification was revised in consensus. As summarized in Table 1 [6], CO-RADS categories range from 0 to six, where 0 indicates that scans are incomplete or of insufficient quality, and six indicates proven COVID-19, indicated by a positive RT-PCR test for virus-specific nucleic acid. As expected, category six was not included in this study because no RT-PCR test was performed in that time frame. The CT findings were not redefined according to the Fleischner Society glossary, as they were only classified according to the CO-RADS classification.

**Table 1:** CO-RADS categories with corresponding level of suspicion for pulmonary involvement in COVID-19 (adapted from [6])

	Level of suspicion for pulmonary involvement of COVID-19	Summary
CO-RADS 0	Not interpretable	Scan technically insufficient for assigning a score
CO-RADS 1	Very low	Normal or non-infectious
CO-RADS 2	Low	Typical for other infections but not COVID-19
CO-RADS 3	Equivocal/unsure	Features compatible with COVID-19, but also other diseases
CO-RADS 4	High	Suspicious for COVID-19
CO-RADS 5	Very high	Typical for COVID-19

### Clinical and Radiological Data Recording

For each patient included in the study, date of birth and date of the CT scan were recorded in a dedicated database. For patients with CT findings classified as CO-RADS 3 or higher categories in 2020, the following clinical chemistry data and clinical symptoms, if available, were recorded: proven infectious disease other than COVID-19 (such as influenza virus, bacteria, and so on); C-reactive protein (CRP); D-dimer; international normalized ratio (INR); oxygen saturation; fever; cough; fatigue; onset of symptoms and date of clinical indication to perform the CT scan.

### Pathological specimen screening

RNA extraction: RNA was extracted from patients included in the aforementioned cohort which agreed to be analyzed for the presence of SARS-CoV-2 infection and for which a cytological sample had been collected at the same time as the CT scan and stored in the archives of the Cantonal Institute of Pathology. Two different kits were used for RNA extraction, depending on the type of cytological material preserved. RNA was extracted either from cytoblocks starting from 2 sections of 10 µm each, using the RNe-asy® FFPE Kit (Qiagen, Chatsworth, CA, USA), following the manufacturer`s instructions, or from cytological smears that were directly subjected to RNA extraction starting from archival Papanicolaou (PAP) test or Periodic acid–Schiff (PAS) colored single section using the RNeasy® Mini Kit (QIAGEN) following the QIAGEN protocol.

The obtained RNA was quantified by a spectrophotometer (Nanodrop 1000, Witec, Littau, CH).

SARS-CoV-2 detection: The presence of the SARS-CoV-2 nucleic acids was detected using the CoviDetectTM (PentaBase, Odense, Denmark) real-time-quantitative polymerase chain reaction (RT-qPCR) assay. The regions evaluated with this assay are those encoding for two SARS-CoV-2 nucleocapsid proteins (N1 and N2) and a region for ex-traction control (RNP, Human RNase P). N1 probe is labeled with the fluorophore FAMTM, N2 with HEXTM and RNP with Cy5TM.

For each sample, regardless of the initial concentration, 5 µl of RNA were aliquoted into CoviDetectTM ready-to-use tubes.

Positive and negative controls provided by the CoviDetectTM kit were extracted using both the aforementioned RNA extraction methods and included in the RT-qPCR analysis. In addition, a blank sample was added to each experiment.

### Statistical analysis

A formal statistical analysis was not possible, due to the lack of true positive and negative cases defined by a gold standard. Therefore, descriptive statistics including the number of findings for each search term, the number of records for each CO-RADS category in 2020 and 2019, the most common clinical indications in CO-RADS 3 and 4 categories, the etiology of pneumonitis in patients classified as CO-RADS 3 or 4, responses to the questionnaires and main clinical data of patients with pneumonitis of unknown origin classified as CORADS 3 or 4, the number of chest CT scans performed in the first two months of 2020 and 2019, with relative comparison, were reported.

## Results

Between January and February 2020, 860 chest CT reports included one or more of the search terms, divided into the following groups: ground-glass n = 185; opacity n = 266; infiltrate n = 199; pneumonitis n = 114; consolidation n = 96. After exclusion of redundant examinations, either performed as follow-up of the CT findings over time or including in the same report more than one term chosen for the selection, the final cohort included 536 patients (mean age 68; standard deviation 14). The CT findings were distributed as follows: ground-glass n = 151 (28%); opacity n = 179 (33%); infiltrate n = 121 (22%); pneumonitis n = 47 (8%); consolidation n = 38 (7%).

In the same time interval of 2019, 670 CT reports included one or more of the search terms. After exclusion of redundant examinations, the final cohort counted 345 patients (mean age 70; standard deviation 13). The CT findings were distributed as follows: ground-glass n = 77 (22%); opacity n = 186 (53%); infiltrates n = 24 (6%); pneumonitis n = 16 (4%); consolidation n = 42 (12%).

Considering ground-glass and infiltrates together, the increase in these findings was considerable in 2020 (50%), compared to 2019 (30%).

**Fig. 1:**
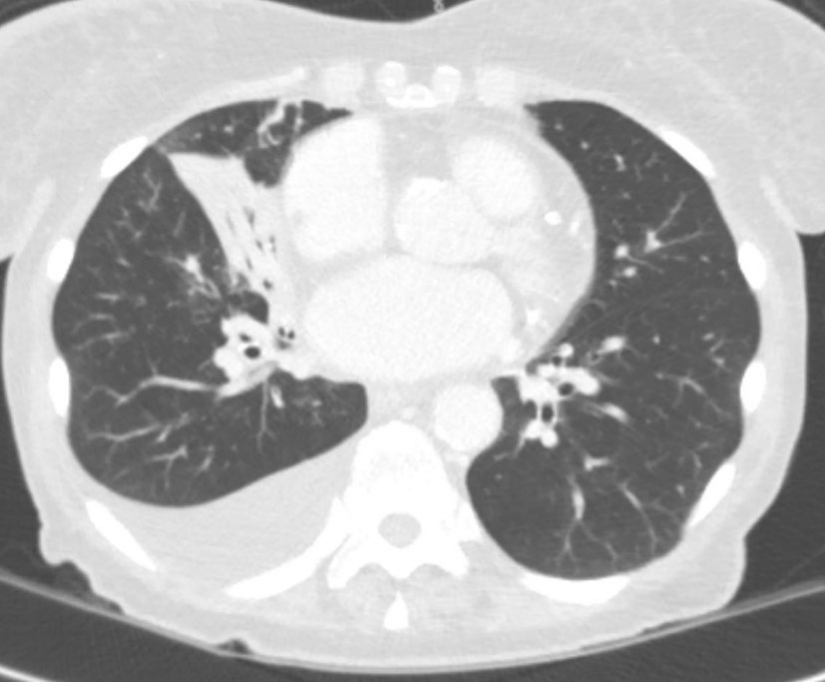
Axial CT image shows consolidation with air bronchogram in the right middle lobe and a right pleural effusion. Although the presence of these findings, the scan was categorized as CO-RADS 0 because it did not include the entire chest and therefore considered insufficient for assigning a score

**Fig. 2:**
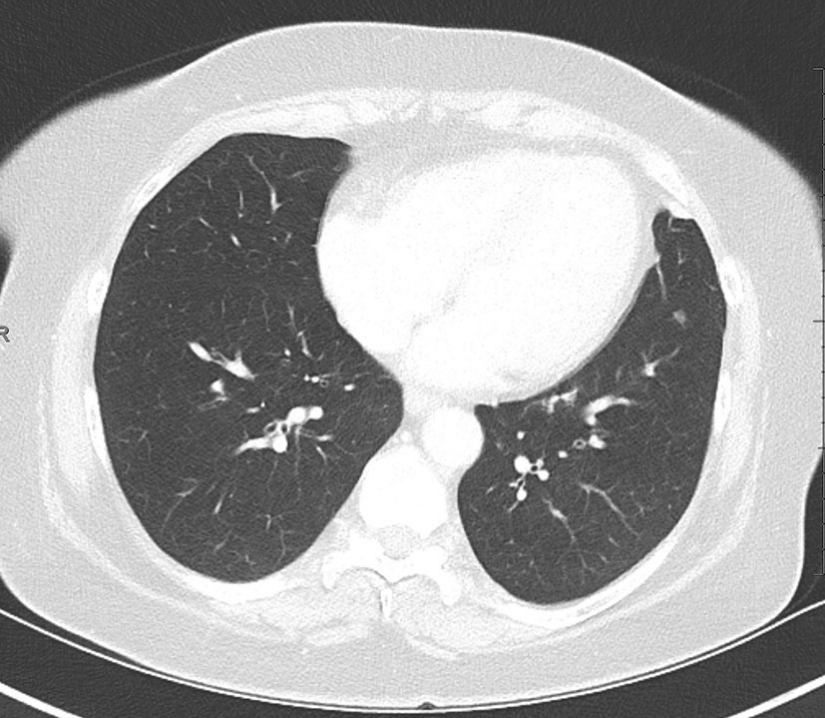
Axial CT image shows a 5 mm partially solid nodule in the left lower lobe that was in follow-up in a patient with a previous renal cell carcinoma. No other remarkable findings were found in the chest CT scan. This exam was categorized as CO-RADS 1, because the finding was clearly non-infectious

**Fig. 3:**
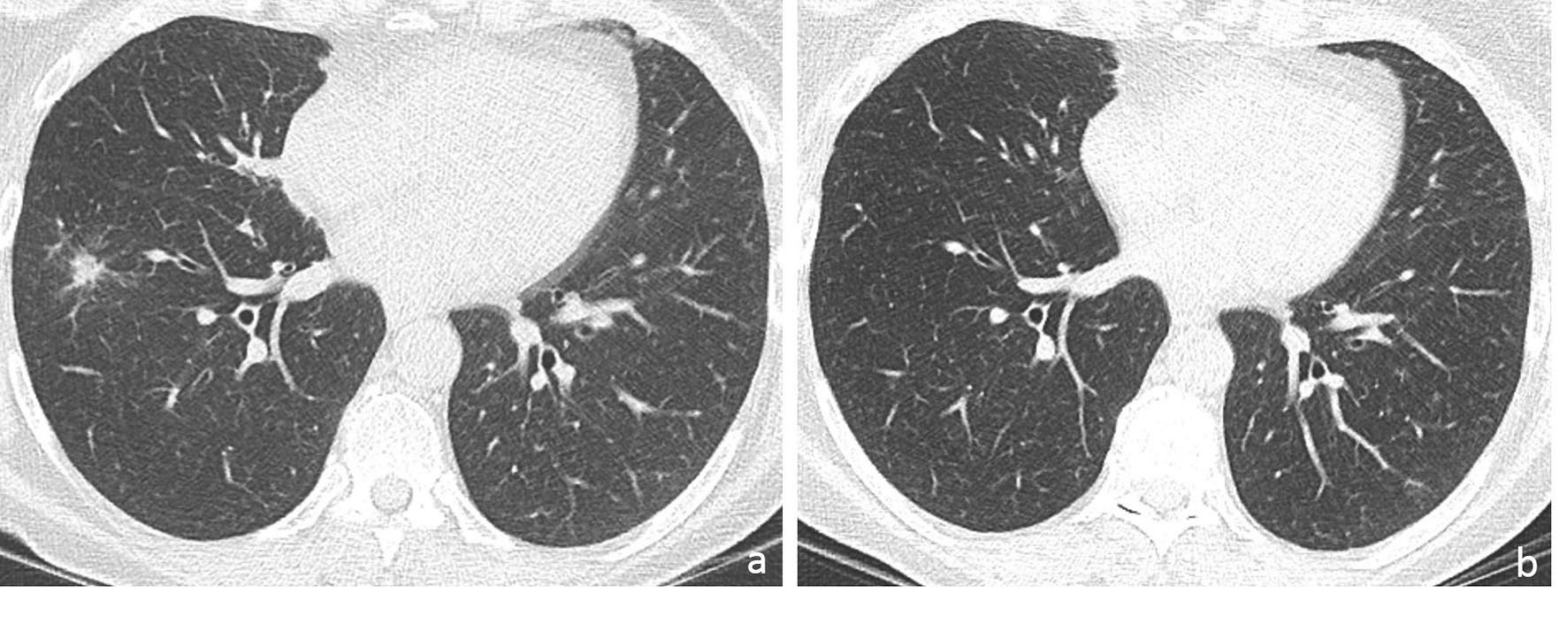
Axial CT image (a) shows a solid nodule with halo sign in the right lower lobe that was categorized as CO-RADS 2 for its typical appearance for other infectious disease (likely bacterial), as demonstrated by its complete disappearance after antibiotic therapy (b)

The final CT records according to the CO-RADS classification in the two time frames analyzed are summarized in Table 2. Examples of CO-RADS categories 0, 1, and 2 are shown in Figs. 1, 2, and 3, respectively.

**Table 2:** Number of final records for each CO-RADS category

	CO-RADS 0	CO-RADS 1	CO-RADS 2	CO-RADS 3	CO-RADS 4	CO-RADS 5
2020	21	250	217	34*	10*	0
2019	7	111	182	42	3	0

*After the exclusion of patients that refused their consent

The most common clinical indications to perform a CT scan in CO-RADS 3 and 4 categories (n = 44) in the first two months of 2020 are shown in Table 3. In these patients the onset of symptoms fell as maximum 10 days before the CT scan, with clinical indication to perform the scan no more than 48 h before.

**Table 3:** Clinical indication to perform the CT scan for the CO-RADS 3 and 4 categories (January 1—February 24, 2020)

Clinical indication	CO-RADS 3 (*n* = 34)	CO-RADS 4 (*n* = 10)
Pulmonary embolism	5	3
Infiltrates	5	1
Oncologic follow-up	5	1
Dyspnea, cough, temperature	8	5
Control after a previous pneumonitis	4	0
Others	7	0

The etiology of pneumonitis in patients classified as CO-RADS 3 or 4 was different from COVID-19 in 14/44 patients (Table 4).

**Table 4:** Etiology of the pneumonitis for the CO-RADS 3 and 4 categories (January 1–February 24, 2020)

Etiology	CO-RADS 3	CO-RADS 4	TOTAL
Infectious disease (other than COVID-19)	5	7	12
Other cause (non-infectious)	2	0	2
Unknown	27	3	30

**Fig. 4:**
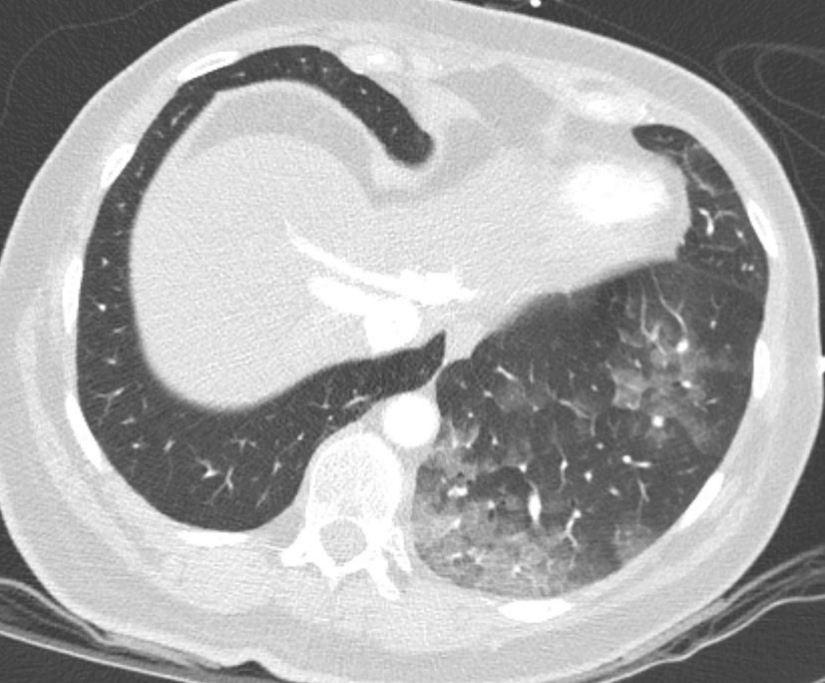
Axial CT image shows ground-glass opacities in the right upper lobe, together with smooth interlobular septal thickening without pleural effusion in the absence of other typical CT findings compatible with COVID-19, classified as CORADS 3

**Fig. 5:**
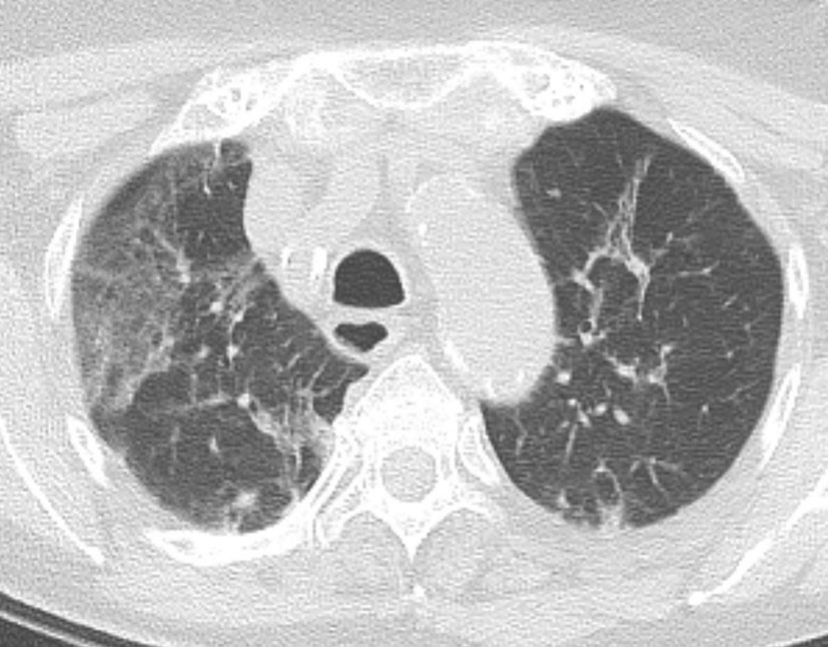
Axial CT image shows unilateral ground-glass opacities in the left lower lobe without consolidations close to the visceral pleural surfaces, classified as CORADS 4

Among the 30/44 patients with pneumonitis of unknown origin: 27 had CT findings categorized as CORADS 3 (Fig. 4) and three as CORADS 4 (Fig. 5). Among patients categorized as CO-RADS 3 and 4 in 2020 (n = 44), ten died in the subsequent 6 months. Two had a diagnosis of COVID-19 between March and April 2020, and one of them died subsequently. Among the ten patients dead in the subsequent 6 months, four died because of progression of an oncological disease; three because of a septic shock; one for a heart attack; one for complications of a severe hepatopathy; one for Covid-19 disease.

Two patients (CORADS 3) declared travel in high-risk areas; 14 showed elevated levels of CRP; four patients (three CORADS 3 and one CORADS 4) showed elevated levels of INR. The most common symptoms were cough (n = 11) and fever (n = 5) (Table 5). No patient referred symptoms such as ageusia or anosmia to the physician.

**Table 5:** Responses to the questionnaires, main serologic data and symptoms of patients with pneumonitis of unknown origin, classified as CORADS 3 or 4

	CO-RADS 3 (*n* = 27)	CO-RADS 4 (*n* = 3)	TOTAL (*n* = 30)
Travels in high-risk areas (Yes)	2	0	2
Serologic test for COVID-19	0	0	0
CRP elevated	13	1	14
D-dimer elevated	4	1	5
INR elevated	3	1	4
Low oxygen saturation	11	1	12
Fever	4	1	5
Cough	10	1	11
Fatigue	4	0	4

The overall number of chest CT scans performed in the first two months of 2020 was 1158, with an 11% increase with respect to the same time interval of 2019 (1049 scans). A comparable increase (+ 12%) was demonstrated also in the subgroup of CT scans acquired without contrast medium administration (448 in 2020 versus 399 in 2019).

Among the 44 patients classified as CO-RADS 3 and 4, nine had pathological specimens collected between January 1 and February 24, 2020, and six consented to use of their specimen to search for the presence of COVID-19. RNA was extracted from cytoblocks in three cases and from cytological smears in the other three cases. In all these six cases, the CoviDetectTM assay failed to identify the presence of SARS-CoV-2 genes: therefore, these patients were classified as COVID-19 negative cases.

## Discussion and Conclusion

This study demonstrates that in the first two months of 2020 about 30 patients had lung infiltrates of unknown origin, with CT appearance compatible with COVID-19 infection, three of which categorized as CORADS 4 (high level of suspicion for COVID-19 related pneumonitis). Though, the performance of CO-RADS classification in a setting with low or unclear prevalence of the COVID-19 disease has an ambiguous diagnostic performance and none of the cases that we could test on pathological specimens had confirmation of the infection at pathology. The number of chest CT scans without contrast medium showed a relative increase of 12% in the first two months of 2020, compared to the same period of 2019.

The presence of sporadic cases of COVID-19 infection before the declaration of the pandemic was hypothesized in other countries. For example, Deslandes et al. reported a case of a patient hospitalized for hemoptysis in an intensive care unit in Paris (France), in December 2019, in which a retrospective analysis on the stored nasopharyngeal swab confirmed the diagnosis of SARS-CoV-2 infection [9]. Similarly, Apolone et al. demonstrated the presence of antibodies in blood samples of subjects recruited in a lung cancer screening program in Milan, (Italy), already in early September 2019 [8]. Authors from Harvard University recorded a considerable increase of hospital traffic in the Wuhan region, according to satellite imagery, and COVID-19 symptoms–related queries in search engines, already since autumn 2019 [10]. However, a recent review published on Lancet considered most of this evidence preliminary and not yet independently verified [11].

COVID-19 presents with CT findings that partially overlap with other diseases, mainly viral infections, but it may also show characteristic features seen less frequently in other settings [12]. As such, it is not surprising that among the 44 CT scans classified as CO-RADS 3 and 4, 14 had an ascertained etiological nature from other infective agents. In our study, the suspicion of COVID-19 related pneumonia was based on CT findings, according to the CO-RADS classification.

The presence of ten cases classified as CORADS 4 in a low-prevalence context and without PCR testing can be considered similar to the first scenario described in the multinational consensus statement of the Fleischner Society. Indeed, this scenario addresses the case of a patient presenting with mild respiratory features (possibly consistent with COVID-19 infection), very low pretest probability of infection (that would be low in our series), and no significant resource limitations. In this scenario, imaging was advised as a good baseline for future comparison, to establish manifestations of important comorbidities and to influence the intensity of monitoring for clinical worsening [13]. Nonetheless, it has been demonstrated that radiologists showed poor diagnostic accuracy when evaluating a sample containing equal percentages of three different types of pneumonia, with a mean accuracy of 70% for COVID-19 pneumonia and 68% for both influenza pneumonia and organizing pneumonia. This low specificity of CT findings was particularly evident in clinical settings where there are substantial proportions of patients with potential causes of organizing pneumonia, such as ongoing cancer therapy or autoimmune conditions [Garrana].

A recent systematic review of thoracic imaging tests for the diagnosis of COVID-19, including 31 studies and 8014 participants, demonstrated that the sensitivity of chest CT ranged from 57.4% to 100%, and specificity ranged from 0% to 96.0%. The pooled sensitivity of chest CT in suspected COVID-19 participants was 89.9% (95% CI 85.7 to 92.9) and the pooled specificity was 61.1% (95% CI 42.3 to 77.1) [13]. All the above-mentioned studies demonstrate that CT can be considered a reliable diagnostic tool, in the absence of RT-PCR. However, it is also known that in a setting of low or unclear prevalence of the disease, as in our setting, the performance of the CO-RADS classification is quite low because of the low power for differential diagnosis of COVID-19 and other pneumonias typical of the winter season [14]

Although these conflicting references, in order to increase our diagnostic performance in the absence of a gold standard, in this study we applied the CO-RADS classification to the lung CT findings. Indeed, it has been demonstrated that the use of a standardized reporting system can significantly improve the specificity of CT results [15–19]. In this regard, Gross et al. demonstrated a sensitivity of 90%, a specificity of 91%, a positive predictive value of 72%, a negative predictive value of 97%, and an accuracy of 91% for CT scan findings to predict the presence of a COVID-19 infection, when the CO-RADS classification was used [20].

In our series, 30 patients showed CT features compatible with COVID-19 pneumonitis, 27/30 classified as CO-RADS 3, indicating that the CT features were possibly due to COVID-19 but not clearly typical, and 3/30 classified as CO-RADS 4, indicating that the CT appearance was likely due to COVID-19. Among the latter, one showed elevated CRP, elevated D-dimer level, low oxygen saturation, cough and fatigue. Unfortunately, pathological material was not available for these 3 latter patients.

A CT scan without contrast medium is usually requested so as to look for lung infiltrates [21]. In order to further explore the hypothesis of a higher number of suspected cases of pneumonitis in the time frame under examination, we compared the number of chest CT scans without contrast medium performed in the first two months of 2020 and 2019, and we found a number 12% higher in January and February 2020 compared to 2019. Among these, the relative percentage of ground-glass opacities and infiltrates showed an increase in 2020 compared to 2019 (28% and 22% in 2020 and 22% and 6% in 2019, respectively).

Nonetheless, a study published in January 2021, demonstrated that the incidence of lethal acute respiratory distress syndrome and pulmonary embolism was uniformly low between October 2019 and February 2020, and that all autopsy cases analyzed by means of SARS-CoV-2 RT-PCR yielded negative results, thus suggesting the absence of early lethal community spread of COVID-19 in Basel (Switzerland) before March 2020 [22].

Our data show that between January 1 and February 24, 2020 there were CT findings compatible with the COVID-19 pneumonia; nonetheless, since the CO-RADS classification performs better in the presence of a high prevalence of the disease (which is not the setting we studied), and it is not possible to perform a swab RT-PCR test a posteriori, nor are there data about the duration of serological antibodies response, the only way to assess if the virus was already circulating before February 2020 was to make a pathological assessment on the available specimens collected before February 2020. Of all our patients, a cytological sample was available in six cases and all of them turned out to be COVID-19 negative by RT-PCR. Such a result does not necessarily indicate that none of the cases were infected by COVID-19 for several reasons: in the analysis, only a few cells and surrounding material were evaluated, and the virus could have been absent in the analyzed material but present in other parts of the respiratory tract; alternatively, the viral load might have been too low for the assay: in support of this hypothesis, two patients, classified as CO-RADS3, displayed a positive RT-PCR assay some weeks after the CT scan. Unfortunately, cytological material from these two patients was not available for the analysis at the time of the CT scan.

This study has some limitations. The main one is the lack of an RT-PCR test, because before the first official case, the RT-PCR was neither requested nor available, and in a retrospective study this data is not available. However, in order to overcome this limitation and to increase the specificity of the CT findings’ interpretation, we used the CO-RADS classification and we asked the patients with suspicious findings for information about the pretest probability (travels in high-risk zones), and for any RT-PCR or serological test results. Another limitation is that our conclusions are based on the analysis of CT scans of patients admitted to our Hospital, which is not the only one in Canton Ticino. However, it is the main public hospital in our area and the potential presence of other suspicious cases from other hospitals could only support our hypothesis. An additional limitation is that our search of CT scans was based on the radiology information system, and this might have led to losing some cases, compared to a search including the chest CT scans from the PACS. However, by using the search terms specified in the text, we retrieved all CT scans with and without the presence of the pre-specified radiological signs, therefore we may assume that we included all the suspicious cases. Finally, the increased number of chest CT scans might be related to the trend of the worldwide increase in CT scans. However, this trend has slowed over time, going from an 11.6% annual percentage increase among adults in 2000–2006 to 3.7% among adults in 2013–2016 [23]; therefore an 11% increase in 1 year seems too high to be associated with this trend.

In conclusion, this study demonstrates that about 30 patients showed CT findings compatible with COVID-19 related pneumonitis in Canton Ticino in the 2 months preceding the first ascertained positive case, but where it was possible to assess the presence of the infection on pathological specimens, it was not found. Furthermore, although we have proved a relative increase in numbers of chest CT scans performed for pneumonitis in the first two months of 2020 compared to 2019, this may still be related to seasonal pneumonias.
